# How argumentation theory can inform assessment validity: A critical review

**DOI:** 10.1111/medu.14882

**Published:** 2022-07-25

**Authors:** Benjamin Kinnear, Daniel J. Schumacher, Erik W. Driessen, Lara Varpio

**Affiliations:** ^1^ Department of Pediatrics University of Cincinnati College of Medicine Cincinnati Ohio USA; ^2^ School of Health Professions Education (SHE) Maastricht University Maastricht The Netherlands; ^3^ School of Health Professions Education Faculty of Health Medicine and Life Sciences of Maastricht University Maastricht The Netherlands; ^4^ Uniformed Services University of the Health Sciences Bethesda Maryland USA

## Abstract

**Introduction:**

Many health professions education (HPE) scholars frame assessment validity as a form of argumentation in which interpretations and uses of assessment scores must be supported by evidence. However, what are purported to be validity arguments are often merely clusters of evidence without a guiding framework to evaluate, prioritise, or debate their merits. Argumentation theory is a field of study dedicated to understanding the production, analysis, and evaluation of arguments (spoken or written). The aim of this study is to describe argumentation theory, articulating the unique insights it can offer to HPE assessment, and presenting how different argumentation orientations can help reconceptualize the nature of validity in generative ways.

**Methods:**

The authors followed a five‐step critical review process consisting of iterative cycles of focusing, searching, appraising, sampling, and analysing the argumentation theory literature. The authors generated and synthesised a corpus of manuscripts on argumentation orientations deemed to be most applicable to HPE.

**Results:**

We selected two argumentation orientations that we considered particularly constructive for informing HPE assessment validity: New rhetoric and informal logic. In new rhetoric, the goal of argumentation is to persuade, with a focus on an audience's values and standards. Informal logic centres on identifying, structuring, and evaluating arguments in real‐world settings, with a variety of normative standards used to evaluate argument validity.

**Discussion:**

Both new rhetoric and informal logic provide philosophical, theoretical, or practical groundings that can advance HPE validity argumentation. New rhetoric's foregrounding of audience aligns with HPE's social imperative to be accountable to specific stakeholders such as the public and learners. Informal logic provides tools for identifying and structuring validity arguments for analysis and evaluation.

## Abstract 2

In #HPE we often conceptualize validity as an argument. But what is argumentation? From what guiding principles might HPE draw? @Midwest_MedPeds @DrDanSchumacher @erikwdriessen @LaraVarpio review argumentation theory to explore these questions.

## 1 INTRODUCTION

Many modern health professions education (HPE) scholars frame assessment validity as a form of argumentation: A claim about a specific interpretation and use of assessment data.^1^, ^2^, ^3^, ^4^ However, HPE scholars have yet to delve into the philosophical, theoretical, or practical aspects of argumentation that undergird validation. What are purported to be validity arguments are often merely the listing of evidence without a guiding framework to evaluate, prioritise, or debate their merits. This is akin to holding a courtroom trial while only knowing how to organise the evidence, but not knowing how the argument should be structured, who will evaluate the argument, nor how they will evaluate it. We contend that it is vital for HPE to develop a deeper understanding of argumentation theory to support our current understandings and uses of validity.

This is not to say that important insights into validity have not already been generated by HPE scholars. Far from it. Our field's understanding of validity has progressed thanks to the work of many HPE researchers. For instance, Cook, Hatala, and Downing have helped bring argument‐based validity frameworks, such as those of Messick and Kane,^5^, ^6^ into the HPE assessment sphere.^1^, ^2^, ^3^, ^7^, ^8^, ^9^, ^10^, ^11^, ^12^, ^13^, ^14^ St. Onge and Young have helped map out the current state of our field's understanding of validity.^4^, ^15^, ^16^, ^17^ Govaerts, Schuwirth, van der Vleuten, and others have progressed our fields view of validity in the context of programmatic assessment.^18^, ^19^, ^20^, ^21^, ^22^, ^23^, ^24^, ^25^, ^26^ It is thanks to scholars like these, and others, that HPE has evolved to conceptualise validity as argument. And yet, HPE's use of contemporary validity frameworks lack an explicit description of the audiences, structures, and evaluation standards for validity arguments. This leaves important questions unanswered: How should validity arguments be evaluated and by whom? How should validity arguments be structured? Not knowing the answers to these questions leaves our field ill‐equipped to fully embrace the validity‐as‐argument paradigm.

In this critical review of the literature, we offer a description of argumentation theory—tailored to the HPE audience—articulating the unique insights it can offer to HPE assessment, and presenting how different approaches to argumentation can help us reconceptualize the nature of validity in generative ways. We present two different orientations within argumentation theory and discuss how each creates different ways of understanding, structuring, and evaluating validity arguments. To make these abstract theories more tangible, we apply these approaches to an HPE‐relevant case (Box 1). Via this case discussion, we highlight how argumentation theories can help advance our assessment efforts. To frame our review, we first offer definitions of validity arguments and argumentation theory.

Box 1 **This case represents a real‐world example of the importance of argumentation paradigms in HPE assessment validity. The case is referenced throughout the Results and Discussion sections**

Case study – A PGME programme transitioning to time‐variable promotion decisions

A post‐graduate medical education programme (PGME) has developed a robust programme of assessment over several years. Programme leaders have collected validity evidence for summative decisions about learner performance via literature review and new validation studies, and are hoping to transition to a time‐variable training approach. Rather than being promoted to lesser degrees of supervision on a time‐based schedule, summative promotion decisions will solely determine when and how learners progress through the programme. When engaging important stakeholders (e.g. learners, PGME leaders, departmental leaders, accreditation officials, certifying bodies) about this potential change, the programme is universally asked, “How do you know your summative promotion decisions are defensible?” In other words, “What is your argument that you can make the right promotion decision?” Before the programme could begin making their argument, they had several questions that needed to be answered. How should their argument be structured? By what standard would it be evaluated? Which audience(s) would levy judgement?

How can new rhetoric inform this case?

The programme leaders recognise that their validity arguments needs to resonate with each stakeholder group; therefore, their argument must to respond to many different expectations. The programme leaders first identify which stakeholders are most important to serve as audiences, attempt to understand their values, and iteratively construct, evaluate, and refine their validity argument until it is acceptable to each stakeholder gorup. Accrediting and certifying bodies are interested in the outcomes and consequences of this specific PGME programme. Therefore, the programme's validity argument includes evidence that patients cared for by graduates receive high‐quality care. In contrast, learners are concerned about equity and fairness in assessment practices. Therefore, the programme's validity argument includes quantitative and qualitative data on how different learner identity groups (e.g. race, ethnicity, gender identity, disability status) are assessed and whether there is evidence of bias against any groups.

How can informal logic inform this case?

Programme leaders collect significant amounts of validity evidence, but initially do not have it organised into a cogent argument for stakeholders to evaluate. Using Toulmin's model, the programme leaders signpost claims, data, warrants, rebuttals, and other salient elements, facilitating easier analysis by the audience. Programme leaders and stakeholders agree that the validity arguments will be evaluated in terms of relevance, acceptability, and sufficiency, and that Toulmin's model will be used to identify any aspects of the argument that are deemed inadequate.

When is an argument complete?

Both stakeholder groups found the programme's initial validity argument to be inadequate, asking for additional evidence and clarification on different argument aspects. Programme leaders worked iteratively with stakeholders to collect and evaluate evidence until both groups found the argument to be acceptable. Programme leaders could then focus resources on engaging other stakeholder groups in similar validity argumentation processes.

### 1.1 Validity arguments within and beyond HPE

Validity is not about assessment scores but rather the interpretations and uses of those scores.^27^, ^28^, ^29^, ^30^ Validation is the process of developing an argument to support those interpretations and uses.^29^ Validity arguments are comprised of claims—i.e. assertions about the phenomena engaged with—that connect to form a defensible chain of reasoning. For instance, we make claims about how competence is defined,^31^ how it can be observed,^32^ and how it can be assessed.^33^ However, these claims are not statements of fact; instead, they are assertions that can be debated, accepted, or refuted. If the claims within a validity argument are viewed as objective, static, and inactive declarations, it is because the argumentation strategies embedded therein have become so pervasively used and accepted that we no longer recognise them as contestable. The validity argument claims upon which HPE assessment decisions rest are merely assertions steeped in argumentation and so are up for debate.

The concept of *validity as argument* has existed and evolved for decades.^34^, ^35^, ^36^ Although many HPE scholars operate using Messick's or Kane's conceptualization of validity argumentation,^1^, ^3^, ^26^, ^37^, ^38^, ^39^, ^40^, ^41^, ^42^, ^43^ scholars outside HPE present other approaches.^44^ Bachman advocates validation work should entail an *assessment use argument*, which is comprised of an *assessment validity argument* linking assessment performance to an interpretation and an *assessment utilisation argument* linking interpretations to a decision.^45^, ^46^ Kane similarly proposes an *interpretation/use argument* that explicitly states the inferences and assumptions ingrained within use of assessment scores.^6^, ^47^ Mislevy describes an *evidence‐centreed design* approach to validation in which the arguments underpinning assessment are integrated into the design and implementation of assessment systems.^48^, ^49^ These three non‐HPE scholars all employ argument structures put forth by Stephen Toulmin,^27^, ^29^, ^45^, ^48^, ^49^, ^50^, ^51^ but they only superficially address the underlying argumentation orientation utilised for validation. Unfortunately, Toulmin's structures—much less argument orientations—have yet to enter the discourse of HPE validation. This omission generates problems for HPE. Embedded within HPE's validity arguments are assumptions and ways of thinking that we often fail to recognise and critique. This failure risks assuming that the validity arguments are objective truths and not structures for reasoning that bring certain ideologies to life. In other words, the arguments entrenched in our assessment decisions and in our conceptualizations of their validity must be made explicit so that we can deliberately consider when and how each decision is valid. Argumentation theory can help us achieve that clarity.

### 1.2 Argumentation theory

Argumentation theory is not a single unified theory; instead, it is a field of study that draws from multiple disciplines (e.g. Logic, Speech, Linguistics, Philosophy, Psychology, and Law) to grapple with the production, analysis, and evaluation of argumentative discourse (spoken or written).^52^, ^53^ Several orientations exist, with scholars from a wide range of academic disciplines adding unique threads to the tapestry of argumentation theory. Van Eemeren et al. provide a definition of argumentation that includes several aspects that are key to argumentation theories:

Argumentation is a verbal and social activity of reason aimed at increasing (or decreasing) the acceptability of a controversial standpoint for the listener or reader, by putting forward a constellation of propositions intended to justify or refute the standpoint before a rational judge.^52^

This conceptualization highlights that argumentation is an endeavour (*activity*) that relies on language (*verbal*) being exchanged between people (*social*). This communication is goal oriented (*aimed*); the goal is to convince an audience (single *listener or reader* or groups of *listeners or readers*) to agree (*increase acceptability*) or disagree (*decrease acceptability*) with a particular viewpoint or interpretation (*standpoint*). Importantly, argumentation exists in social situations where the viewpoint being espoused is disputable (*controversial*) by an audience. Therefore, arguments (*constellations of propositions*) are created to present a particular interpretation of the viewpoint (*justify or refute the standpoint*) that will convince the audience (*rational judge*) to align themselves with the arguer. Without a shared conception of processes and standards, argumentation is futile.^52^

Many different theories of argumentation propose how arguments could be realised. Some foreground the use of deductive reasoning,^54^ whereas others accentuate the power of dialogue.^55^ Some stress normative standards,^56^ whereas others emphasise rhetorical aims.^57^ Furthermore, argumentation scholars debate and revise each of these theories, offering different interpretations. Clearly, a review of this entire body of scholarship would not readily offer practical insights for advancing HPE's thinking about validity. However, a more targeted review of argumentation theories that can offer important considerations and concrete applications that could be harnessed by HPE's assessors and assessment researchers. Therefore, we selected theories and scholarly interpretations thereof that could be usefully applied to validity argumentation in HPE.

## 2 METHODS

Our investigation asked: How can argumentation theory help us conceptualise the nature of HPE assessment validity in new and generative ways? To answer this question, we conducted a critical review. Critical reviews are not intended to produce generalizable truths^58^ like systematic reviews. Rather, critical reviews are rooted in a constructivist ontology and epistemology^59^ and “draw on literature and theory from different domains to re‐envision current ways of interpreting the problem.”^60^ Instead of aiming to synthesise all knowledge relevant to a particular topic, the research team conducts a critical review and subjective interpretation of a body of literature. The research team thus “acts as a research instrument, using their perspective to appraise and interpret the literature uncovered.”^60^ This approach aligns with our goal of describing a selection of argumentation theories that can advance HPE by examining the assumptions and preconceptions around assessment validity. We followed the five‐step critical review process detailed by Kahlke et al to generate a corpus of theories and to analyse that corpus.^60^ We engaged in iterative cycles of focusing (i.e. constructing/revising the research aims); searching (i.e. targeted exploration for sentinel perspectives in argumentation theory); appraising (i.e. assessing manuscripts for relevance to our aims); sampling (i.e. determining manuscripts potential to offer insights into our research); and analysing (i.e. assessing each theory's applicability to validity argumentation).

**FIGURE 1:**
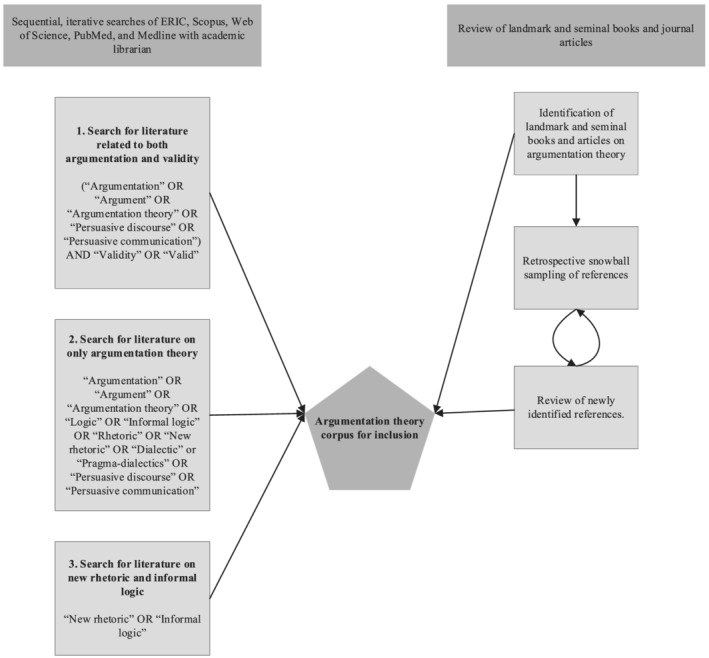
An iterative process for building an argumentation theory corpus for analysis

Our search strategy (Figure 1) began by locating seminal works in the field of argumentation theory by identifying highly cited peer‐reviewed journal articles that were written by authors considered leaders in the field of argumentation theory. Given that argumentation theory sits in the humanities, we recognised that, although peer‐reviewed journal articles would be informative, we would also have to broaden our scope to books and textbooks since these are highly valued forms of dissemination in this domain. We reviewed these manuscripts and mapped the leading strains of thought in argumentation theory that addressed theoretical and/or practical aspects of argumentation from several points in the field's history. We used our expertise in argumentation and HPE assessment validity to “appraise papers for inclusion based on their sense of a source's relevance to the research question and the value added by the information it contains.”^60^ In parallel to our review of seminal works, we worked with an academic librarian to search ERIC, Scopus, Web of Science, PubMed, and Medline databases because they index a wide range of literatures—i.e. both journal articles and books—addressing humanities‐ and education‐related publications. We searched for highly cited books and peer‐reviewed journal articles that were not included in our original corpus. We used snowball sampling retrospectively (i.e. investigating references listed in seminal works) to identify additional key references.

BK and LV reviewed the corpus to independently create lists of the pre‐eminent theories and scholars that have shaped the field of argumentation. These lists were not intended to be comprehensive, but rather to capture the most influential voices and viewpoints within argumentation. We then compared and resolved these into a single list through consensus discussions. We reviewed sources from the corpus that aligned with the consensus list to determine which argumentation theories were most applicable to HPE. Specifically, we considered which theories would provide beneficial insights to HPE scholars engaged in assessment validation, both in the context of practical validation work and broader scholarly endeavours. Table 1 provides a summary of this work, presenting prominent orientations within argumentation theory which we considered, though some we did not fully discuss in our findings.

**TABLE 1:** Prominent argumentation theories and their relevance to health professions education

Orientation	Description	Influential scholars	Applicability to validity arguments in health professions education (HPE)
Formal logic^61^, ^62^	Arguments are abstracted from their real‐world environments and presented in a standard form of premises and conclusions (called syllogisms) for purposes of deductive reasoning. The goal of logic is to ensure that conclusions necessarily flow from premises.	Aristotle Antoine Arnauld Pierre Nicole John Stuart Mill George Boole	Formal logic statements are disconnected from real‐world circumstances, which is antithetical to the highly contextual complex adaptive systems that constitute HPE. Therefore, we deem this theory to be poorly aligned with HPE's context.
Informal logic	Arguments are evaluated with real‐world contextual factors included. There is an emphasis on identifying, structuring, and evaluating arguments against normative standards such as relevance, sufficiency, acceptability, and defensibility.	J. Anthony Blair Trudy Govier Ralph Johnson Charles Hamblin Howard Kahane Stephen Toulmin Douglas Walton John Woods	Informal logic provides tools for identifying and structuring arguments for analysis and evaluation without removing context. HPE validity arguments are often complex and not clearly articulated. Therefore, we decided that informal logic could provide useful approaches to HPE validity argumentation.
New rhetoric	Arguments are aimed to persuade and gain an audience's adherence to the arguer's claim. Audience values and norms define the acceptability of an argument. Audiences can be particular or universal and should be clearly identified by arguers.	James Crosswhite Lucie Olbrechts‐Tyteca Chaim Perelman	New rhetoric foregrounds the imperative to persuade audiences. Given that HPE is comprised of several different audiences each with unique expectations vis a vis validity, we decided that new rhetoric could offer important insights to HPE.
Formal dialectics^63^, ^64^, ^65^	Argumentation centres around the resolution of (mostly verbal) disputes between specific interlocutors. A highly‐structured normative system of rules guides critical dialogue should unfold. The goal is to defeat the other interlocutor while adhering to the discursive rules.	Roland Barth Charles Hamblin Erik C. W. Krabbe	Formal dialects and pragma‐dialectics are a poorer fit given the highly structured rules of engagement and the oppositional nature of arguer and audience (i.e. the goal is to “win” the argument).
Pragma‐dialectics^52^, ^66^	Argumentation centres around the resolution of disputes between specific interlocutors. Ten discursive rules govern argumentation, which unfolds in four stages: confrontation, opening, argumentation, and concluding. The goal is to defeat the other interlocutor while adhering to the discursive rules.	Rob Grootendorst Frans van Eemeren	

### 2.1 Reflexivity

The critical review research team “acts as a research instrument, using their perspectives to appraise and interpret the literature uncovered.”^60^ With this goal in mind, our unique perspectives, areas of expertise, and personal backgrounds informed the synthesis activity. Our team consisted of medical education scholars with diverse interests and experiences. Two team members (BK and DS) are clinician educators who actively engage in HPE research. The study's primary researcher (BK) immersed himself in the argumentation theory literature as part of his PhD in HPE; he also has expertise in competency‐based medical education (CBME), learner assessment, and validity. The other clinician educator (DS) has expertise in CBME and assessment validity. ED is a PhD‐trained researcher with expertise in learner assessment, validity, and CBME. The senior researcher on this paper (LV) is a PhD‐trained researcher whose graduate work focused on argumentation theory, rhetoric, and professional communication. Her insights helped the team to identify significant scholars and theories in argumentation and to reflect on how these scholars and theories aligned with other approaches to validity espoused in HPE. Team research discussions often involved debates about which argumentation theories could be usefully applied to concerns about validity in HPE.

Methodological reflexivity also guided our research. By reviewing the theories from several authors and the common assumptions held within and across schools of argumentation theory, we selected theories we thought held the most generative possibilities for HPE assessment validity. We sought to include a broad representation of argumentation theories, but the guiding principle primarily informing our selections was: Can this theory substantially help us understand validity arguments in the HPE context?

## 3 RESULTS

In Table 1, we outline key aspects of five prominent theories of argumentation, along with our assessments of each theory's applicability to HPE's validity arguments. Through our iterative cycles of analysis, we determined that two orientations—new rhetoric and informal logic—offered particularly useful insights into HPE assessment validity. This is not to say that other argumentation orientations are without value for HPE; instead, based on our expertise, we suggest that new rhetoric and informal logic are especially applicable to assessment validity in our field. We first review these orientations, then focus on three aspects of argumentation that we believe are relevant to validity argumentation in HPE: audience, argument structure, and evaluation standards.

### 3.1 New rhetoric

In the mid‐20th century two philosophers, Chaim Perelman and Lucie Olbrechts‐Tyteca, developed a theory of argumentation that relied more on value judgements than empirical proof.^67^, ^68^, ^69^, ^70^ New rhetoric was so named because of similarities to Aristotle's rhetoric, particularly the central importance Aristotle placed on audience and persuasion in argumentation.^61^, ^71^ In new rhetoric, the goal of argumentation is not the demonstration of absolute truth but rather to gain an audience's acceptance of a claim being put forth by an arguer.^53^, ^72^ Argument validity is dependent upon audience persuasion. Arguers must account for and adapt to an audience's values and beliefs, thereby linking validity to the ability to convince an audience.^57^ Validity exists as a matter of degree since an audience might be partially persuaded towards an arguer's claim. In this sense, new rhetoric does not provide an external normative framework for determining argument validity; instead, it relies on an audience to use their own values and norms to determine what is acceptable and plausible. Persuasion does not remove the need for rational argument analysis; it places rationality in the context of an audience's values. Importantly, persuasion is not meant as a way to deceive or manipulate,^73^ but rather to consider and apprize a particular audience's principles and anticipated standards as evaluative criteria.

Given the centrality of an audience's values and standards to validity, arguers must make explicit their intended audience. Perelman and Olbrects‐Tyteca distinguish between arguing to a *particular* audience and a *universal* audience. A particular audience consists of a real‐world person or group (or representative thereof) to whom an arguer can address their argument.^74^ Although the particular audience may only be a subset or sample of a larger group, the argument still occurs using a concrete person's or group's values as the standard. In contrast, the universal audience is conceptual, conceived by the arguer as a group holding the values and ideals of competent and reasonable people. Thus, with a universal audience, the arguer plays a role in determining the values and standards of validity based on their notion of a reasonable audience. There is debate over the specific roles of particular and universal audiences in new rhetoric^74^, ^75^, ^76^, ^77^; however, with both particular and universal audience, the arguer's goal is securing audience adherence. New rhetoric compels arguers to explicitly identify their audience, and incentivizes arguers to possess knowledge and understanding of their audience.

Practical implications of new rhetotic for assessment practices in HPE include the need for programme leaders to tailor their validity arguments to meet the needs of each group they are engaging. When addressing accrediting and certifying bodies, arguments might emphasise validity evidence focused on outcomes and consequences of training. When addressing learners, arguments might emphasise equity and fairness in assessment practices.

### 3.2 Informal logic

Informal logic provides procedures and standards for identifying (via structures) and evaluating (via normative standards) arguments that occur in real‐world, social environments and public discourse while still valuing logical coherence between premises and conclusions.^56^, ^78^ Informal logic is a broad umbrella term covering differing approaches to determining argument validity.^79^, ^80^ Before the advent of this theory, formal logic was applied to real‐world arguments in a sterile, decontextualized way. Many informal logic scholars rejected this context‐free approach because it was fraught with potential distortions that rendered evaluating real‐world argumentation impossible. Informal logic was developed to account for the messy, implicit, incomplete nature of real‐world argumentation through identification of argument structures without removal from contextual factors. Once an argument's structure is contextually understood, informal logic offers multiple standards for determining the strength of inferences in the argument.^56^ One approach relies on the criteria of *relevance*, *acceptability*, and *sufficiency* to evaluate arguments.^81^, ^82^ Relevance demands adequate relationship between premises (i.e. claims or propositions), conclusions inferred from these premises, and the overall argument. Acceptability relates to the truth or plausibility of premises as determined by the arguer, audience, and critical community. Sufficiency requires that evidence supporting an argument be appropriate in type, quantity, and use. Other approaches for determining the strength of an argument in informal logic include excluding fallacies^83^, ^84^ (i.e. errors of reasoning), using counterexamples, and formulating argumentation schemes.^85^ No one approach is singularly correct for determining an argument's validity; instead, informal logic foregrounds argument strength and cogency as the goal of argumentation.

Philosopher Stephen Toulmin profoundly influenced informal logic with his 1958 book *The Uses of Argument* ^86^ in which he provided a framework for organising argument structures to facilitate analysis and evaluation.^87^, ^88^ In Toulmin's model, arguments are deconstructed into unique component parts of a larger cohesive unit. A *claim* is a standpoint or opinion on a particular matter taken by an arguer which serves as the point of departure for argumentation. Claims are supported by evidence which Toulmin labels *data*. However, an arguer must elaborate on how or why particular data support the stated claim. This elaboration is known as a *warrant*, which provides justification for use of particular data to substantiate a claim. In other words, a warrant provides a bridge between a claim and its supporting data.^52^ The data‐warrant‐claim unit forms the backbone of Toulmin's argumentation structure, though there are other components such as *backings* (evidence to support a warrant), *qualifiers* (limitations or restrictions on the universality of a claim), and *rebuttals* (pre‐emptive or reactive responses to counterarguments).

In Toulmin's model, validity hinges on demonstrating that the warrant adequately justifies the leap from data to claim. He believed that similar argument structures could be used regardless of the relevant discipline, or *field*.^86^ Thus, argument structure and the general procedure by which arguments could be analysed are *field independent*. However, Toulmin believed that many aspects of validity are *field dependent*, meaning that arguments should be evaluated using the norms, values, and criteria of the relevant field. To that end, Toulmin uses terms such as *defensible*, *acceptable*, and *sound* when describing validity. The audience who levies such determinations can be a specific person or group participating in the argument or an onlooker who is not involved in the argument but can make a judgement nonetheless.

Practical implications of informal logic for assessment practices in HPE include training programme leaders clearly signposting the claims, data, warrants, and backings of their argument, allowing stakeholders to more clearly identify the inferences needing scrutiny to determine the validity of time‐variable promotion decisions. Accepted claims then become data or warrants for other arguments related to the programme of assessment informing promotion decisions. Thus, a clearly labelled web of argumentation could be formed to support time‐variable decisions. This web would be field dependent, meaning that it would be evaluated using acceptability and soundness criteria that are relevant in HPE, though not necessarily in other disciplines.

## 4 DISCUSSION

We contend that informal logic and new rhetoric are theories of argumentation that hold promise to deepen our understanding of validity argumentation in HPE. Both approaches acknowledge the importance of context in argumentation and align with Zumbo's conceptualization of validity as contextualised and pragmatic.^89^, ^90^ We are not asserting that HPE's validity practices should be wedded to any particular orientation; instead, we propose that our field could benefit from a useful, reciprocal relationship between HPE validation practices and established argumentation theories.^91^ For example, Cook and Hatala provide an excellent practical guide for undertaking validation work in HPE using Kane's framework.^8^ We suggest enhancing this guide by including considerations of to whom an argument should be directed (audience), how to organise and signpost arguments within and between inferences (structure), and/or how an argument should be evaluated (standards). With regard to the latter, Cook and Hatala use language that seems to invoke both new rhetoric and informal logic, noting that validity arguments attempt to “persuade others” while also noting that the “relevance, quality, and breadth” of evidence is important. Just as there are few definitive boundaries in the field of argumentation, with scholars within and across traditions debating the meaning, scope, and utility of various orientations, we suggest that HPE should not silo itself into one argumentation orientation. HPE researchers can draw from various traditions based on which will be most helpful to move our field's understanding of validity forward. Therefore, we believe HPE would benefit from using aspects of both new rhetoric and informal logic to deepen our understanding of audience, argument structure, and evaluation standards in validity argumentation.

### 4.1 New rhetoric to define HPE validity argumentation's audience

A new and growing discourse in HPE is that of “validity as social imperative,”^4^, ^17^ which emphasises accountability to society and learners. In other words, HPE validity arguments centre on the needs of our stakeholder audiences. Informal logic recognises the importance of audience in argumentation, but not to the same degree as new rhetoric.^92^ New rhetoric places audience values at the centre of argumentation, and may help us better satisfy the social imperative that HPE is being called to fulfil by better engaging both stakeholders and the broader HPE community.

As mentioned previously, new rhetoric arguments can be directed towards a particular audience or a universal audience. For HPE to adopt a new rhetoric approach to validity argumentation, we would need to better define our audiences. Currently, many formal HPE validity arguments are made in the form of peer‐reviewed publications, with journal reviewers, editors, and readers serving as audiences. Journal readers may be the most important audience in the publication world. However readers represent a form of noninteractive audience^93^ (with the exception of the very few who write letters or commentaries), which limits the degree to which their values can be known or their judgements shared. Reviewers and editors could serve as a particular audience representing other HPE scholars, or they could be considered a representation of a universal audience because they are most likely to represent competent and reasonable people with sufficient knowledge to levy informed judgements. They could, in fact, serve both roles. However, currently reviewers and editors are not necessarily making explicit judgements on whether a validity argument is acceptable or plausible, and certainly not within the context of a given institution or programme. Rather, they are making a determination of whether a manuscript, in toto, moves the broader scholarly discussion forward in the HPE community. Reviewers and editors could, in fact, find a validity argument to be inadequate but still recommend publication because the manuscript provides some benefit to the journal's readers, perhaps in terms of innovative thinking, methodological developments, or novel conclusions. Thus, new rhetoric would compel reviewers and editors—were they to be identified as a particular audience—to explicitly judge the plausibility of validity arguments submitted for publication, perhaps with a way to signal their judgement to readers upon publication.

The limited audience interaction that occurs with publication would oblige HPE to expand validity argumentation beyond the pages of journals. HPE has several stakeholders in assessment decisions including learners, programme leaders, accreditors, certifiers, institutions, and payors. Many of these stakeholders may never read the articles in which validity evidence relevant to their context is published. Assessment designers would need to ensure that arguments are plausible to these particular audiences, likely via mechanisms that do not involve peer‐reviewed publication. Whereas informal logic presents argument as a product, new rhetoric entreats argumentation to be an activity, or service, that an arguer develops with an audience.^53^ This activity‐based orientation aligns with Cook and Hatala's description of validation as a process,^8^ and with the concept of co‐production, which is gaining traction HPE.^94^, ^95^ In co‐production, the consumers' knowledge, experience, and opinions influence the creation of a service rather than passive consumption of a product.^96^, ^97^ Adopting a lens of new rhetoric could the coproduction of arguments over time with validity arguers and audiences (i.e. stakeholders) working to best meet the needs of those who HPE aims to serve.

### 4.2 Informal logic to clarify HPE validity argumentation structure

HPE scholars regularly organise validity evidence using frameworks from Messick and Kane,^2^, ^3^ but do not make full argument structure explicit. Indeed, Kane invokes Toulmin's argumentation structure (warrants, data, backings, rebuttals),^27^, ^29^, ^47^, ^50^ though this model has mostly appeared in other fields such as language testing.^98^ Toulmin's model exists as a cryptid in HPE, rarely (if ever) surfacing within our corpus. Kane's framework requires arguers to clearly state an intended interpretation and use of assessment data,^6^, ^29^ which serves as the argument claim. However, rather than simply using Kane's chain of inferences, HPE scholars could make explicit the full structure of their arguments to augment analysis, particularly for high‐stakes decisions that require significant scrutiny. In fact, there are likely to be several arguments embedded within Kane's chain of inferences,^28^ which may go overlooked without explicit and structured reporting. Complex validity arguments that are likely to be found in programmatic assessment could be mapped out, showing how an established claim becomes data or a warrant for a subsequent argument in a longer chain of argumentation. Though there is debate amongst validity scholars as to how much structure the validation process should have,^44^ currently HPE has no clear or agreed‐upon approach for organising, identifying, or signposting validity arguments. Often what are labelled “arguments” are collections of data that lack an explicit argument structure. Toulmin's model could help to present, interpret, prioritise, and argue the evidence to stakeholders.

### 4.3 Informal logic or new rhetoric to elucidate standards for evaluating validity arguments

Neither informal logic nor new rhetoric claim to provide a universal truth, but they could inform when seemingly unending validation efforts have reached defensible stopping points (at least until new arguments are put forth or new audiences are considered). New rhetoric seeks persuasion and audience adherence. Informal logic seeks argument cogency, often in terms of relevance, sufficiency, and acceptability as defined by the arguer's discipline or field. Both lenses would work well within HPE; therefore, choosing which to use may depend on one's philosophical worldview. Informal logic may align well with post‐positivist worldviews,^99^, ^100^ whereas new rhetoric likely aligns better with constructionist or critical realist views.^101^, ^102^ However, informal logic and new rhetoric are not restricted to any specific worldview; each will manifest in different ways when employed with different philosophical beliefs. Given the heterogenous philosophical worldviews in HPE, is it reasonable to expect one argumentation orientation to fit all arguers and audiences? Likely not. But we should explicitly acknowledge our argumentation paradigms when undertaking validity work, and specifically consider our audience's as well. Doing so can help avoid misunderstanding of worldviews and argumentation paradigms that could cause conflict in HPE validation work.^103^ Tavares et al described a *compatibility principle* in assessment as “the obligation to recognize that different philosophical positions can exist between and within assessment plans and that these positions commit assessment designers to particular ideas and assumptions.”^104^ We believe the compatibility principle also applies to the philosophies of argumentation woven into HPE validity.

### 4.4 Remaining challenges of using argumentation in HPE assessment validation

Though we believe informal logic and new rhetoric are useful orientations for HPE validity argumentation, several challenges must still be addressed. First, little is known about which argumentation orientations resonate with HPE stakeholders such as learners, programme leaders, accreditors, and certifiers. Future research could begin to unearth the latent argumentation assumptions that these groups may carry. Second, catering to the values of multiple audiences could make validity argumentation a daunting task in a new rhetoric paradigm. Each stakeholder group likely has different values and understandings of assessment and validity. It is unclear if educators can develop a validity argument that is unique to each group, or a single argument that is persuasive and acceptable to all. Third, we must consider and study the impact of infusing informal logic or new rhetoric into validation on hegemony and equity within HPE, particularly with regard to audience values and evaluative standards. Addey et al call for “democratic spaces” in which “legitimately diverse arguments and intentions can be recognized, considered, assembled, and displayed.”^105^ Creating such democratic spaces can ensure that all stakeholder voices are heard. Finally, although we believe Toulmin's argumentation model could allow for improved analysis and evaluation of HPE validity arguments, empirical studies are needed to test this hypothesis. Until the HPE community explores these limitations and challenges, assessment validity argumentation will remain a black box for programmes such as the one in our case study.

### 4.5 Limitations

Though our author group selected informal logic and new rhetoric as most useful for HPE validity argumentation, there are several other argumentation orientations that we did not select (a small selection is presented in Table 1). We acknowledge that other scholars might find these other orientations more useful to HPE, particularly if they value discursive structure (formal dialectics and pragma‐dialectics) or have positivist worldviews (formal logic). We also acknowledge that given the long and rich history of argumentation theory, it was impossible for us to fully review every orientation and approach therein.

### 4.6 Conclusion

HPE scholars have published robust validity evidence^13^, ^38^, ^39^, ^40^, ^106^ for various assessment decisions organised within the commonly used frameworks of Messick^5^ or Kane.^27^ Although these provide excellent examples of how to organise validity evidence, they do not explicitly describe who should evaluate the evidence (audience), what structure the argument (not just the evidence) should take, and what criteria should be used for evaluation. This omission propagates the notion that evidence equals argument and that a validity judgement has been rendered by simply laying out the evidence. In this manuscript, we have detailed how informal logic and new rhetoric can help advance HPE's ongoing work with validity in assessment. Each theory offers affordances for transforming ambiguous, inert HPE assessment validity evidence into clearer, animate validity arguments.

## CONFLICTS OF INTEREST

The authors have no competing interests to report.

## AUTHOR CONTRIBUTIONS

All authors contributed to the conception and design of this critical review. BK performed the bulk of the literature review. BK and LV led the analysis, with input from ED and DS on which argumentation orientations are most relevant to HPE. BK wrote the initial draft of the paper. All authors were responsible for revising the paper, contributing to choices on which orientations to include in our final manuscript, and which aspects of argumentation to include. All authors approved the final manuscript.

## ETHICAL APPROVAL

Not applicable.

## DISCLAIMER

This work was prepared by a military or civilian employee of the US Government as part of the individual's official duties and therefore is in the public domain. The opinions and assertions expressed herein are those of the author(s) and do not necessarily reflect the official policy or position of the Uniformed Services University or the Department of Defense.

## References

[1] Cook DA . When I say … validity. Med Educ. 2014;48(10):948‐949. doi:10.1111/medu.12401 25200015

[2] Cook DA , Beckman TJ . Current concepts in validity and reliability for psychometric instruments: theory and application. Am J Med. 2006;119(2):e167‐e116. doi:10.1016/j.amjmed.2005.10.036 16443422

[3] Cook DA , Brydges R , Ginsburg S , Hatala R . A contemporary approach to validity arguments: a practical guide to Kane's framework. Med Educ. 2015;49(6):560‐575. doi:10.1111/medu.12678 25989405

[4] St‐Onge C , Young M , Eva KW , Hodges B . Validity: one word with a plurality of meanings. Adv Health Sci Educ Theory Pract. 2017;22(4):853‐867. doi:10.1007/s10459-016-9716-3 27696103

[5] Messick S . Validity. In: Linn R , ed. Educational Measurement. New York: Macmillan Publishing; 1989:13‐103.

[6] Kane M . The argument‐based approach to validation. School Psychol Rev. 2013;42(4):448‐457. doi:10.1080/02796015.2013.12087465

[7] Cook DA , Lineberry M . Consequences validity evidence: evaluating the impact of educational assessments. Acad Med. 2016;91(6):785‐795. doi:10.1097/ACM.0000000000001114 26839945

[8] Cook DA , Hatala R . Validation of educational assessments: a primer for simulation and beyond. Adv Simul (Lond). 2016;1(1):31. doi:10.1186/s41077-016-0033-y 29450000 PMC5806296

[9] Downing SM . Validity: on the meaningful interpretation of assessment data. Med Educ. 2003;37(9):830‐837. doi:10.1046/j.1365-2923.2003.01594.x 14506816

[10] Downing SM , Haladyna TM . Validity threats: overcoming interference with proposed interpretations of assessment data. Med Educ. 2004;38(3):327‐333. doi:10.1046/j.1365-2923.2004.01777.x 14996342

[11] Downing SM , Yudkowsy R . Assessment in Health Professions Education. New York, NY: Routledge; 2009. doi:10.4324/9780203880135

[12] Cook DA , Kuper A , Hatala R , Ginsburg S . When assessment data are words: validity evidence for qualitative educational assessments. Acad Med. 2016;91(10):1359‐1369. doi:10.1097/ACM.0000000000001175 27049538

[13] Hatala R , Cook DA , Brydges R , Hawkins R . Constructing a validity argument for the Objective Structured Assessment of Technical Skills (OSATS): a systematic review of validity evidence. Adv Health Sci Educ. 2015;20(5):1149‐1175. doi:10.1007/s10459-015-9593-1 25702196

[14] Cook DA , Zendejas B , Hamstra SJ , Hatala R , Brydges R . What counts as validity evidence? Examples and prevalence in a systematic review of simulation‐based assessment. Adv Health Sci Educ. 2014;19(2):233‐250. doi:10.1007/s10459-013-9458-4 23636643

[15] St‐Onge C , Young M . Evolving conceptualisations of validity: impact on the process and outcome of assessment. Med Educ. 2015;49(6):548‐550. doi:10.1111/medu.12734 25989399

[16] Young M , St‐Onge C , Xiao J , Lachiver EV , Torabi N . Characterizing the literature on validity and assessment in medical education: a bibliometric study. Persp Med Educ. 2018;7(3):182‐191. doi:10.1007/s40037-018-0433-x PMC6002290 29796976

[17] Marceau M , Gallagher F , Young M , St‐Onge C . Validity as a social imperative for assessment in health professions education: a concept analysis. Med Educ. 2018;52(6):641‐653. doi:10.1111/medu.13574 29878449

[18] Wilby KJ , Govaerts MJ , Dolmans DH , Austin Z , van der Vleuten C . Reliability of narrative assessment data on communication skills in a summative OSCE. Patient Educ Couns. 2019;102(6):1164‐1169. doi:10.1016/j.pec.2019.01.018 30711383

[19] de Jonge LP , Timmerman AA , Govaerts MJ , et al. Stakeholder perspectives on workplace‐based performance assessment: towards a better understanding of assessor behaviour. Adv Health Sci Educ. 2017;22(5):1213‐1243. doi:10.1007/s10459-017-9760-7 PMC5663793 28155004

[20] van der Vleuten CP , Schuwirth LW . Assessing professional competence: from methods to programmes. Med Educ. 2005;39(3):309‐317. doi:10.1111/j.1365-2929.2005.02094.x 15733167

[21] van der Vleuten CP , Schuwirth LW , Driessen EW , et al. A model for programmatic assessment fit for purpose. Med Teach. 2012;34(3):205‐214. doi:10.3109/0142159X.2012.652239 22364452

[22] Govaerts M , van der Vleuten CP . Validity in work‐based assessment: expanding our horizons. Med Educ. 2013;47(12):1164‐1174. doi:10.1111/medu.12289 24206150

[23] Driessen EW , Overeem K , Van Tartwijk J , Van Der Vleuten CP , Muijtjens AM . Validity of portfolio assessment: which qualities determine ratings? Med Educ. 2006;40(9):862‐866. doi:10.1111/j.1365-2929.2006.02550.x 16925636

[24] Schuwirth LW , Van der Vleuten CP . Programmatic assessment: from assessment of learning to assessment for learning. Med Teach. 2011;33(6):478‐485. doi:10.3109/0142159X.2011.565828 21609177

[25] Bok HG , de Jong LH , O'Neill T , Maxey C , Hecker KG . Validity evidence for programmatic assessment in competency‐based education. Persp Med Educ. 2018;7(6):362‐372. doi:10.1007/s40037-018-0481-2 PMC6283777 30430439

[26] McGill DA , van der Vleuten CP , Clarke MJ . Construct validation of judgement‐based assessments of medical trainees' competency in the workplace using a “Kanesian” approach to validation. BMC Med Educ. 2015;15(1):237. doi:10.1186/s12909-015-0520-1 26715145 PMC4696206

[27] Kane MT . An argument‐based approach to validity. Psychol Bull. 1992;112(3):527‐535. doi:10.1037/0033-2909.112.3.527

[28] Wools S , Sanders P , Eggen T . Evaluation of validity and validation by means of the argument‐based approach. Eval Valid Validation Means Argument‐Based Approach. 2010;(1):1000‐1020. doi:10.3280/CAD2010-001007

[29] Kane MT . Validating the interpretations and uses of test scores. J Educ Meas. 2013;50(1):1‐73. doi:10.1111/jedm.12000

[30] LeBaron Wallace T . An argument‐based approach to validity in evaluation. Evaluation. 2011;17(3):233‐246. doi:10.1177/1356389011410522

[31] Frank JR , Mungroo R , Ahmad Y , Wang M , De Rossi S , Horsley T . Toward a definition of competency‐based education in medicine: a systematic review of published definitions. Med Teach. 2010;32(8):631‐637. doi:10.3109/0142159X.2010.500898 20662573

[32] Kogan JR , Hatala R , Hauer KE , Holmboe E . Guidelines: the do's, don'ts and don't knows of direct observation of clinical skills in medical education. Perspect Med Educ. 2017;6(5):286‐305. doi:10.1007/s40037-017-0376-7 28956293 PMC5630537

[33] Holmboe ES , Sherbino J , Long DM , Swing SR , Frank JR , Collaborators IC . The role of assessment in competency‐based medical education. Med Teach. 2010;32(8):676‐682. doi:10.3109/0142159X.2010.500704 20662580

[34] Cronbach LJ . Five perspectives on validity argument. Test Validity. 1988;3‐17.

[35] House ER . The Logic of Evaluative Argument. Center for the Study of Evaluation, UCLA Graduate School of Education; 1977.

[36] Cronbach LJ , Meehl PE . Construct validity in psychological tests. Psychol Bull. 1955;52(4):281‐302. doi:10.1037/h0040957 13245896

[37] Hodwitz K , Tays W , Reardon R . Redeveloping a workplace‐based assessment program for physicians using Kane's validity framework. Can Med Educ J. 2018;9(3):e14‐e24. doi:10.36834/cmej.42286 PMC6104320 30140344

[38] Hess BJ , Kvern B . Using Kane's framework to build a validity argument supporting (or not) virtual OSCEs. Med Teach. 2021;1‐6(9):999‐1004. doi:10.1080/0142159X.2021.1910641 33834949

[39] Tavares W , Brydges R , Myre P , et al. Applying Kane's validity framework to a simulation based assessment of clinical competence. Adv Health Sci Educ. 2018;23(2):323‐338. doi:10.1007/s10459-017-9800-3 29079933

[40] Kinnear B , Kelleher M , May B , et al. Constructing a validity map for a workplace‐based assessment system: cross‐walking Messick and Kane. Acad Med. 2021;96(7S):S64‐S69. doi:10.1097/ACM.0000000000004112 34183604

[41] Tavares W , Gofton W , Bhanji F , Dudek N . Reframing the O‐SCORE as a retrospective supervision scale using validity theory. J Grad Med Educ. 2022;14(1):22‐24. doi:10.4300/JGME-D-21-00592.1 35222815 PMC8848889

[42] Hamstra SJ , Cuddy MM , Jurich D , et al. Exploring the association between USMLE scores and ACGME milestone ratings: a validity study using national data from emergency medicine. Acad Med. 2021;96(9):1324‐1331. doi:10.1097/ACM.0000000000004207 34133345 PMC8378430

[43] Hamstra SJ , Yamazaki K . A validity framework for effective analysis and interpretation of milestones data. J Grad Med Educ. 2021;13(2s):75‐80. doi:10.4300/JGME-D-20-01039.1 33936537 PMC8078069

[44] Lavery MR , Bostic JD , Kruse L , Krupa EE , Carney MB . Argumentation surrounding argument‐based validation: a systematic review of validation methodology in peer‐reviewed articles. Educ Meas Issues Pract. 2020;39(4):116‐130. doi:10.1111/emip.12378

[45] Bachman LF . Building and supporting a case for test use. Lang Assess Q. 2005;2(1):1‐34. doi:10.1207/s15434311laq0201_1

[46] Bachman LF . Constructing an assessment use argument and supporting claims about test taker‐assessment task interactions in evidence‐centered assessment design. Measurement‐Lawrence Erlbaum Associates. 2003;1:63‐65.

[47] Kane M . Validating score interpretations and uses: Messick lecture, language testing research colloquium, Cambridge, April 2010. Lang Testing. 2012;29(1):3‐17. doi:10.1177/0265532211417210

[48] Mislevy RJ , Steinberg LS , Almond RG . Focus article: on the structure of educational assessments. Meas Interdiscip Res Persp. 2003;1(1):3‐62.

[49] Mislevy RJ . Validity by design. Educational Researcher. 2007;36(8):463‐469. doi:10.3102/0013189X07311660

[50] Kane M . Validity and fairness. Language Testing. 2010;27(2):177‐182. doi:10.1177/0265532209349467

[51] Mislevy RJ . The case for informal argument. Meas Interdiscip Res Persp. 2012;10(1–2):93‐96. doi:10.1080/15366367.2012.682525

[52] Van Eemeren FH , Grootendorst R , Johnson RH , Plantin C , Willard CA . Fundamentals of Argumentation Theory: A Handbook of Historical Backgrounds and Contemporary Developments. Routledge; 2013. doi:10.4324/9780203811306

[53] Tindale CW . Acts of Arguing: A Rhetorical Model of Argument. SUNY Press; 1999.

[54] Corcoran J . Aristotle's demonstrative logic. Hist Philos Logic. 2009;30(1):1‐20. doi:10.1080/01445340802228362

[55] Krabbe EC , Walton DN . Formal dialectical systems and their uses in the study of argumentation. In: Keeping in Touch with Pragma‐Dialectics; 2011:245‐263. doi:10.1075/z.163.17kra

[56] Blair JA . What is informal logic? In: Reflections on Theoretical Issues in Argumentation Theory. Springer; 2015:27‐42. doi:10.1007/978-3-319-21103-9_2

[57] Perelman C , Olbrechts‐Tyteca L . The new rhetoric: A treatise on argumentation, trans. In: John Wilkinson and Purcell Weaver (Notre Dame). Vol.19. IN: University of Notre Dame Press; 1969.

[58] Grant MJ , Booth A . A typology of reviews: an analysis of 14 review types and associated methodologies. Health Info Libr J. 2009;26(2):91‐108. doi:10.1111/j.1471-1842.2009.00848.x 19490148

[59] Colliver JA . Constructivism: the view of knowledge that ended philosophy or a theory of learning and instruction? Teach Learn Med. 2002;14(1):49‐51. doi:10.1207/S15328015TLM1401_11 11865749

[60] Kahlke R , Lee M , Eva KW . Critical reviews in health professions education research. J Grad Med Educ. PMID: In press. 10.4300/JGME-D-23-00154.1 PMC10150820 37139200

[61] Aristotle JB . The Complete Works of Aristotle. Vol. 2. NJ: Princeton University Press Princeton; 1984.

[62] Boole G . The Laws of Thought. New York (original edition 1854): Dover; 1957.

[63] Barth EM , Krabbe EC . Formal dialectics: instruments for the resolution of conflicts about expressed opinions. Spektator. 1978;7(307):341.

[64] Barth EM , Krabbe EC . From Axiom to Dialogue: A Philosophical Study of Logics and Argumentation. Walter de Gruyter; 2010.

[65] Krabbe EC . Formal systems of dialogue rules. Synthese. 1985;63(3):295‐328. doi:10.1007/BF00485598

[66] Van Eemeren FH , Grootendorst R . Argumentation, Communication, and Fallacies: A Pragma‐Dialectical Perspective. Routledge; 2016. doi:10.4324/9781315538662

[67] Perelman C , Olbrechts‐Tyteca L . Logique et rhétorique. Rev Philos France Let. 1950;140:1‐35.

[68] Perelman C , Olbrechts‐Tyteca L . Rhétorique et philosophie; pour une théorie de l'argumentation en philosophie. Revue de Métaphysique et de Morale. 1953;58(3).

[69] Frank DA , Bolduc M . Lucie Olbrechts‐Tyteca's new rhetoric. Q J Speech. 2010;96(2):141‐163. doi:10.1080/00335631003796685

[70] Crosswhite J . The new rhetoric project. Philosophy & Rhetoric. 2010;43(4):301‐307. doi:10.5325/philrhet.43.4.0301

[71] Van Eemeren FH . Argumentation theory after the new rhetoric. L'analisi Linguistica e Letteraria. 2009;17(1):119‐148.

[72] Frank DA . Argumentation studies in the wake of the new rhetoric. Argumentation Advocacy. 2004;40(4):267‐283. doi:10.1080/00028533.2004.11821612

[73] Paso M . Rhetoric meets rational argumentation theory. Ratio Juris. 2014;27(2):236‐251. doi:10.1111/raju.12043

[74] Sigler J . The new rhetoric's concept of universal audience, misconceived. Argumentation. 2015;29(3):325‐349. doi:10.1007/s10503-015-9349-3

[75] Aikin SF . Perelmanian universal audience and the epistemic aspirations of argument. Philos Rhetoric. 2008;41(3):238‐259. doi:10.5325/philrhet.41.3.0238

[76] Gross AG . Misunderstanding the universal audience. Adv History hetoric. 2019;22(3):290‐302. doi:10.1080/15362426.2019.1671704

[77] Perelman C . The new rhetoric and the rhetoricians: remembrances and comments. Q J Speech. 1984;70(2):188‐196. doi:10.1080/00335638409383688

[78] Blair JA . The “logic” of informal logic. In: Groundwork in the Theory of Argumentation. Springer; 2012:101‐117. doi:10.1007/978-94-007-2363-4_9

[79] Johnson RH . Making sense of “informal logic”. Informal Logic. 2006;26(3):231‐258. doi:10.22329/il.v26i3.453

[80] Walton D , Brinton A . Historical Foundations of Informal Logic. Routledge; 2016. doi:10.4324/9781315253329

[81] Johnson RH , Blair JA . Logical self‐defense. Idea. 2006.

[82] Walton D . Formalizing informal logic. Douglas Walton and Thomas F Gordon, Formalizing Informal Logic, Informal Logic. 2015;35(4):508. doi:10.22329/il.v35i4.4335

[83] Hamblin CL . Fallacies. Tijdschrift Voor Filosofie. 1970;33(1).

[84] Walton D . Informal Logic: A Pragmatic Approach. Cambridge University Press; 2008.

[85] Hitchcock D . Informal logic and the concept of argument. In: Philosophy of Logic. Elsevier; 2007:101‐129. doi:10.1016/B978-044451541-4/50007-5

[86] Toulmin S . The Uses of Argument. Cambridge: Cambridge University Press; 1958.

[87] Hitchcock D , Verheij B . The Toulmin model today: introduction to the special issue on contemporary work using Stephen Edelston Toulmin's layout of arguments. Argumentation. 2005;19(3):255‐258. doi:10.1007/s10503-005-4414-y

[88] Simosi M . Using Toulmin's framework for the analysis of everyday argumentation: some methodological considerations. Argumentation. 2003;17(2):185‐202. doi:10.1023/A:1024059024337

[89] Stone J , Zumbo B . Validity as a pragmatist project: a global concern with local application. In: Trends in Language Assessment Research and Practice; 2016:555‐573.

[90] Zumbo BD . Validity as contextualized and pragmatic explanation, and its implications for validation practice. Paper presented at: The Concept of Validity: Revisions, New Directions and Applications, Oct, 2008 2009.

[91] Kvernbekk T . Argumentation in theory and practice: gap or equilibrium? Informal Logic. 2012;32(3):288‐305. doi:10.22329/il.v32i3.3534

[92] Johnson RH . The role of audience in argumentation from the perspective of informal logic. Philosophy & Rhetoric. 2013;46(4):533‐549. doi:10.5325/philrhet.46.4.0533

[93] Govier T , Hoaglund J . The Philosophy of Argument. Vol. 3. Newport News, VA: Vale Press; 1999.

[94] Dollinger M , Lodge J , Coates H . Co‐creation in higher education: towards a conceptual model. J Market Higher Educ. 2018;28(2):210‐231. doi:10.1080/08841241.2018.1466756

[95] Holmboe ES . Work‐based assessment and co‐production in postgraduate medical training. GMS J Med Educ. 2017;34(5):Doc58. 29226226 10.3205/zma001135 PMC5704603

[96] Elwyn G , Nelson E , Hager A , Price A . Coproduction: when users define quality. BMJ Qual Saf. 2020;29(9):711‐716. doi:10.1136/bmjqs-2019-009830 PMC7467503 31488570

[97] Turakhia P , Combs B . Using principles of co‐production to improve patient care and enhance value. AMA J Ethics. 2017;19(11):1125‐1131. doi:10.1001/journalofethics.2017.19.11.pfor1-1711 29168684

[98] Knoch U , Chapelle CA . Validation of rating processes within an argument‐based framework. Language Testing. 2018;35(4):477‐499. doi:10.1177/0265532217710049

[99] Park YS , Konge L , Artino AR . The positivism paradigm of research. Acad Med. 2020;95(5):690‐694. doi:10.1097/ACM.0000000000003093 31789841

[100] Young ME , Ryan A . Postpositivism in health professions education scholarship. Acad Med. 2020;95(5):695‐699. doi:10.1097/ACM.0000000000003089 32345881

[101] Rees CE , Crampton PE , Monrouxe LV . Re‐visioning academic medicine through a constructionist lens. Acad Med. 2020;95(6):846‐850. doi:10.1097/ACM.0000000000003109 31809294

[102] Ellaway RH , Kehoe A , Illing J . Critical realism and realist inquiry in medical education. Acad Med. 2020;95(7):984‐988. doi:10.1097/ACM.0000000000003232 32101916

[103] MacLeod A , Ellaway RH , Paradis E , Park YS , Young M , Varpio L . Being edgy in health professions education: concluding the philosophy of science series. Acad Med. 2020;95(7):995‐998. doi:10.1097/ACM.0000000000003250 32101927

[104] Tavares W , Kuper A , Kulasegaram K , Whitehead C . The compatibility principle: on philosophies in the assessment of clinical competence. Adv Health Sci Educ. 2020;25(4):1003‐1018. doi:10.1007/s10459-019-09939-9 31677146

[105] Addey C , Maddox B , Zumbo BD . Assembled validity: rethinking Kane's argument‐based approach in the context of International Large‐Scale Assessments (ILSAs). Assess Educ Principles Policy Practice. 2020;27(6):588‐606. doi:10.1080/0969594X.2020.1843136

[106] Hawkins RE , Margolis MJ , Durning SJ , Norcini JJ . Constructing a validity argument for the mini‐clinical evaluation exercise: a review of the research. Acad Med. 2010;85(9):1453‐1461. doi:10.1097/ACM.0b013e3181eac3e6 20736673

